# The miracle of peer review and development in science: an agent-based model

**DOI:** 10.1007/s11192-017-2244-y

**Published:** 2017-03-03

**Authors:** Simone Righi, Károly Takács

**Affiliations:** 1 Department of Agricultural and Food Sciences, Alma Mater Studiorum - University of Bologna, Viale Fanin 50, 40127 Bologna, Italy; 2 MTA TK “Lendület” Research Center for Educational and Network Studies (RECENS), Hungarian Academy of Sciences, Országház utca 30, Budapest, 1014 Hungary

**Keywords:** Peer review, Evolution of cooperation, Reputation, Agent based model, C63, C65, C72, C73

## Abstract

It is not easy to rationalize how peer review, as the current grassroots of science, can work based on voluntary contributions of reviewers. There is no rationale to write impartial and thorough evaluations. If reviewers are unmotivated to carefully select high quality contributions, there is no risk in submitting low-quality work by authors. As a result, scientists face a social dilemma: if everyone acts according to his or her own self-interest, the outcome is low scientific quality. We examine how the increased relevance of public good benefits (journal impact factor), the editorial policy of handling incoming reviews, and the acceptance decisions that take into account reputational information, can help the evolution of high-quality contributions from authors. High effort from the side of reviewers is problematic even if authors cooperate: reviewers are still best off by producing low-quality reviews, which does not hinder scientific development, just adds random noise and unnecessary costs to it. We show with agent-based simulations why certain self-emerged current practices, such as the increased reliance on journal metrics and the reputation bias in acceptance, work efficiently for scientific development. Our results find no proper guidelines, however, how the system of voluntary peer review with impartial and thorough evaluations could be sustainable jointly with rapid scientific development.

## Introduction

Peer review is the fundamental process used by the scientific community to select and to ensure the quality of academic publications (cf. e.g., Alberts et al. 2008; Bornmann 2013; Squazzoni and Takács 2011). Several generations of scientists have contributed high-quality reviews, while only authorship has been credited for academic career. It is not easy to rationalize why researchers provide impartial reviews and constructive advice voluntarily, as they need to sacrifice time that could be used for their own research activities (Bernstein 2013). In consequence, it is a puzzle how the system of peer review can be sustainable at all.

We target the understanding of this puzzle and the consequent foundational problems of scientific development by creating and analyzing a simple agent-based model. The model considers a community of scientists who write and evaluate the papers of each other under different filtering policies of journal editors. Among other agent-based models of peer review, such as Gilbert (1997), Squazzoni and Gandelli (2012), Kovanis et al. (2016), Day (2015) and Bianchi et al. (2017), the main strength of the current study lies in the fact that it targets the motivational trap of reviewers and highlights that the trap also implies free riding behavior in scientific production. We seek solutions within the model that could potentially guide recommendations for editorial policies to ensure the selection of high quality publications.

The puzzle concerned can be described as a double social dilemma game where scientists can choose levels of efforts for reviews and manuscripts (cf. Bianchi et al. 2017; Squazzoni et al. 2013). Given the presence of costs in terms of time and effort, no contribution (sloppy review) is the best reply strategy for reviews. When all reviewers play according to their best reply strategy, the resulting outcome is no scientific control on the quality of submitted papers. The dominant strategy equilibrium of low quality reviews means that submissions need not be of high quality. Due to the immense costs of producing high quality work, authors are best off by submitting poor manuscripts and waiting for the publication of their work by chance. In short, in the lack of explicit sanctions and incentives, low-quality submissions and low-quality reviews form the dominant strategy equilibrium in the double social dilemma of scientific production.

Social dilemmas are difficult to resolve in general, and those that lack clear benefits of mutual cooperation are troublesome in particular. Substantiating and internalizing the values of scientific development that could be considered as the reward of cooperation is therefore crucial. The most evident improvement might come from introducing higher payoffs for cooperation. For instance, a public good reward for overall cooperation can be established. The good standing of a journal is achieved with excellent publication material and public awareness of its high standards of review. This good standing is a public good that benefits all authors who published in the given outlet. When taken into account in academic career, journal metrics, such as the impact factor, serve as indicators of quality—irrespective of having them deserved with hard work or acquiring them undeservedly with luck.

Another straightforward modification of the payoff scheme of the social dilemma is the application of selective incentives (Olson 1965): the allocation of additional benefits for authors of high-quality papers (e.g., promotion and grants, which is current practice) and for reviewers writing high-quality reports (which is rare practice).

Direct (Hamilton and Axelrod 1981; Axelrod 1984) and indirect reciprocity (Boyd and Richerson 1989; Milinski et al. 2001, 2002a, b; Nowak and Sigmund 2005; Semmann et al. 2005; Nowak 2006) are potential solutions to social dilemmas, if the chance of repetition is high and the opportunities for retaliation are in foresight. In the context of peer review, indirect reciprocity can be facilitated, for instance, by rotating the roles of authors, reviewers, and editors (Bravo et al. 2015). The social embeddedness of scientific production further enhances the chance of solving the social dilemma efficiently (Coleman 1986; Barrera 2008). The small world aspect of working in specific fields noted by Newman (2001) and the intertwined network of co-authorship (Bianchi et al. 2017), participation in international project consortia, and hangouts at conferences all reduce the competitiveness and contribute to the pro-social character of reviewing.

Furthermore, once informal communication, gossip, reputation, image scoring, and stratification enter into the structure of the social dilemma, cooperation might emerge and be maintained (Sommerfeld et al. 2007, 2008). Recording of and relying on reputational scores have become a catalyst of cooperation in many areas of life (Dellarocas 2003). Reputation is one of the most important motivations for scientists to work hard, while it is also used as a signal of high quality. When reputation is increasingly taken into account for judging scientific quality, the “rich will get richer”, generating a Matthew effect (Merton et al. 1968; Newman 2001; Squazzoni and Gandelli 2012; Wang 2014; Perc 2014). Earlier achievement and reputational information certainly play an important role in current practice both for editorial and reviewer decisions (Paolucci and Sichman 2014).

Social incentives that are associated with direct and indirect reciprocity and the social embeddedness of peer review might be key mechanisms that rationalize cooperative behavior of reviewers. The importance of social incentives is highlighted by surveys asking for the motivations of reviewers (Malicki et al. 2016; Warne 2016). In fact, monetary incentives might be in conflict with or drive out social incentives when applied to reviewers (Squazzoni et al. 2013; Chetty et al. 2014).

In this paper, we build on the view that the aim of peer review is to ensure the selection of valuable scientific papers for publication (Gilbert 1997). The model emphasizes the costly character of high-quality manuscript submissions and reviewer contributions. We take it as granted that high-quality submissions lead to better science, while reviews impact scientific quality only indirectly. Our model makes radical simplifications on the practical aspects of the peer review system intentionally. This way, we aim at providing a straightforward assessment of the institutional conditions and editorial policies under which authors are directly motivated to produce high-quality submissions and reviewers are motivated to provide high quality reviews. Hence, we are primarily interested in the emergence of cooperation that results in the selection of scientific quality.

The rest of this paper is divided in three sections. In “The model” section, we introduce our agent-based model of scientific work and peer review. We report simulations results in the “Results” section and conclude in the “Conclusion” section.

## The model

### The baseline model of peer review

We build an agent based model which contains *N* scientists writing single-author papers. At each discrete period *t*, each scientist performs both the task of author—by producing one article—and the one of reviewer. Authors and reviewers comprise of an identical set of agents. For the sake of simplicity, we consider a single journal with a single editor, who is not an author or a reviewer in the journal.

Authors can produce low or high quality contributions that they submit to the editor who considers them in random order. The editor selects $\mu$ (set to two in our simulations, as this is the most common practice in most journals) reviewers for each paper chosen uniformly at random^1^ with an upper limit of reviews for each reviewer *k* (the value of *k* is set to 4 without loss of generality, as shown in the robustness check reported in Fig. 14). We assume that reviewers always accept requests, but they do not necessarily invest high effort in performing reviews. It is of their strategic decision to produce a low quality review at low cost (normalized to zero) or a high quality review at high cost (*E*). The former is the best reply strategy of the reviewer if no further incentives are provided. The review is translated into a binary recommendation (accept or reject) which is passed on to the editor. We assume that the recommendation is random with fifty–fifty percent chance of accept or reject in case of low reviewer effort and reflects the quality of the submission perfectly in case of high reviewer effort. The editor’s decision is based on incoming recommendations and it has a binary outcome: accept or reject. We consider a single round of review. Acceptance benefits the author, but benefits the editor (the journal and scientific development in general) only if the submission was of high quality.

The incentive structure and the strategy space of the game are defined as follows. The editor wants to maximize high-quality scientific output in the journal and would like to minimize the number of low-quality articles appearing in the journal. This means that similarly to the model of Bianchi et al. (2017), papers that are accepted and of bad quality are the most harmful for the journal (Squazzoni and Gandelli 2012). These values are used as output measures to evaluate the performance of the entire system in our simulations.

The situation is of asymmetric information (Squazzoni et al. 2013), in which the editor is unable to assess the true quality of submissions or the true effort of reviewers. The true quality of accepted papers is revealed probabilistically after publication (we fix the revelation probability $P_{reveal}=1$ in the simulations reported) and there is no way for the editor to assess the true quality of rejected submissions. The crucial parts of editorial policy are therefore the selection of reviewers who can be trusted for their recommendations and the extent of reliance on reviewer recommendations. Several strategies could possibly be used in order to arrive at a proper conclusion. We vary these editorial strategies in between simulations, because these could be easily translated into policy recommendations.

Keeping a reputational account of scientists is part of possible editorial strategies. Editorial reputations of scientists are improved largely by good publications (named *GP*), degraded even more by bad publications (*BP*) and worsened by rejected submissions (*Rej*). Moreover, the editor is in a privileged position to overview the reviewer activities of scientists. Every good review scientists write (*GR*) increases, while every bad review they write (*BR*) decreases their editorial reputation. The quality of the review is only determined for the editor after publication. Good reviews are those where the paper is revealed of the same quality of what the reviewer said. Bad reviews are those where there is a difference, i.e. where the paper is revealed as bad while the reviewer said that it was good or where the paper was revealed as good, but the reviewer recommended rejection (in case of conflicting reviews). Finally reviews for rejected papers are considered of good quality if they recommended rejection, and of bad quality otherwise. Please note the asymmetric character of reviewer bias: as the high quality of rejected papers is never revealed, rejection is a safer strategy for reviewers (a rejection recommendation can only turn out to be a bad review in case other reviewers recommended publication, the paper has been published, and its true high quality has been revealed). In summary, the editorial reputation of scientist *i* is given as:

1 $$\begin{aligned} REP_i^E = \#GP-\#Rej- \alpha \cdot \#BP+ \gamma \cdot (\#GR-\#BR) \end{aligned}$$

where $\#Rej=\#BN+\#GN$ (the sum of bad and good rejected papers), $0<\gamma \ll 1.$ Parameter $\alpha >1$ represents the relative detrimental effect for the journal reputation of accepting a low quality paper. The parameter $\gamma$ represents instead the relative weight of the agent’s behaviour as referee compared to the one as author. For the sake of our simulations both parameters are set conservatively in order to avoid favouring the emergence of cooperative behaviours. Indeed, $\gamma$ is fixed to 0.10, meaning that the behaviour of the author as reviewer of peers’ papers weights one tenth of its behaviour as author. Similarly $\alpha =2$ in all our simulations meaning that the detrimental effect of publishing a bad paper is twice as important as the positive effect on reputation of the publication of a high quality paper. Small variations around these values do not materially change the results discussed in the “Results” section. Note that, given the specification of Eq. (1), as papers and reviews grow over time $REP_i^E$ can grow or decrease unboundedly. However, the absolute value of authors’ reputation is not salient for editorial decisions, which only uses relative reputations to eventually bias its decisions (as noted in the “Reputation-based selection and journal impact factor” section).

Incoming reviews are assumed to lead to editorial conclusions according to an editorial policy which is assumed to be fixed and not updated within a simulation. We manipulate editorial strategies between the simulations in order to compare the effectiveness of these policies. Editorial strategies differ with regard to the handling of conflicting reviews and relying on author reputations in desk rejection and acceptance. The latter element is only added in the extended model presented in the “Reputation-based selection and journal impact factor” section. In the simulations reported, we consider four editorial strategies with regard to the handling of conflicting reviews. For all of them, if all referees agree, then the editor follows the unanimous advice. In case of *disagreement*, the editor can use one of the following strategies:

- **AP**: Reject the paper (i.e., the editor accepts a paper only if All reviews are Positive);
- **1P**: Accept the paper (i.e. the editor accepts the paper if at least 1 review is Positive);
- **ER**: Follow the advice of one of the referees chosen at random probability proportional to the relative Editorial Reputations of the referees;
- **MR**: Follow the advice of the Most Reputed referee.

Authors have perfect information about the quality of their own submissions. Similarly to the model of Bianchi et al. (2017), authors decide to submit a paper of low quality (at zero cost) or at high quality (at cost *e*). They are best off with the publication of their low-quality papers (*BP*). Thus, their preference order is: $BP> GP> BN > GN$.

When in the role of reviewers, scientists first accept editorial requests to review submissions. We assume that this does not entail any significant costs: the time spent on pushing the “accept” button in an editorial managerial system is negligible and the social costs of being committed to reviewing are counterbalanced by gaining access to papers before their publication. Once papers are assigned to reviewers, they decide to invest low effort (at zero cost) or high effort (at cost *E*) in performing the task. Thus, low effort is the best reply strategy of reviewers.

For the sake of simplicity, we assume that all individual payoffs are in the positive domain. Hence the costs *e* and *E* could be considered as opportunity costs of time that are lost by high effort investments in the given period ($0<E<e$).^2^ Altogether, writing high quality papers as well as writing high quality reviews entail sacrificing valuable research and leisure time for scientists.

We disregard the potential time conflict between writing high-quality papers and reviews. In models of (Bianchi et al. 2017; Kovanis et al. 2016), scientists need to allocate time between submissions and reviewing. In our model, both activities are costly if they are performed well and it is not a necessity that scientists invest high effort in one of them. At the same time, we assume no gain from not being published. A publication always yields a positive return ($V_{acc}=3$) that outweighs the costs of high effort *e*.

In line with the current duality of practices, two cases are compared: single blind and double blind reviews (Seeber and Bacchelli 2017). In case of single blind reviews, reviewers can condition their recommendations on the reputation of the author. In case of double blind reviews, only the editor is able to make decisions based on the reputation of authors. In case of a single blind review system, the reviewer strategy can be conditional on the public reputation of the author. Unlike the editorial reputation of authors, the public reputation of an author *i* depends only on *published* papers and does not severely punishes bad publications:

2 $$\begin{aligned} REP_i^P = \#GP-\#BP \end{aligned}$$

*Agents’ strategies* As reviewers are also authors, the strategies concerning the two aspects of the scientist’ work are bundled and are characterized by the following elements:

- A decision for the production of manuscripts, that can be either:
  - *c*: produce manuscripts of high quality (cooperation)
  - *d*: produce manuscripts of low quality (defection)
- A decision for the production of reviews, that can be either:
  - *C*: produce reviews at high effort (cooperation)
  - *D*: produce reviews at low effort (defection)
  - *Rep*: exercise high effort with a likelihood that is proportional to the public reputation of the author of the paper (cooperation *conditional* on reputation).^3^

Individual strategies are assigned to scientists at the start randomly and in equal numbers for each combination. This results in four bundled strategies for the double blind case and in six bundled strategies for the single blind case. Authors decide according to their strategy types to submit a paper of low quality or at high quality (at high cost), which is sent out for review according to the rules determined by the editorial policy. Reviewers act according to their strategies and provide recommendations of accept or reject to the editor. Papers are selected for publication as a result of the recommendations of the reviewers and the editorial policy.

*The evolution of individual strategies* The evolution of author/reviewer strategies is modeled with a replicator dynamics rule adjusted to a finite population. Scientists tend to adopt strategies that ensured higher average payoffs in the previous time period in the population, while they tend to disregard strategies that resulted in lower payoffs.

The author/reviewer’ payoff at the end of each period *t* is defined as the payoff from accepted papers minus the efforts invested in writing manuscripts and reviews, as: $V_{acc}-e(GP^t+GN^t)- E\cdot \#GR$. The first term $V_{acc}$ is the baseline payoff of publishing a paper and it is set to 3 if the paper produced by this author was published, zero otherwise. The second term is the cost of producing the paper, and is set to $e=1$ if the paper is of high quality (regarless of the fact that it has been published), zero otherwise. The last term accounts for the cost *E* a reviewer has to pay for each paper reviewed with high effort.

Each individual has a probability $P_{evo}=0.01$ of being selected for updating his strategy. If this happens, then his new strategy is selected randomly with a probability proportional to the difference of the given strategy to the average expected payoff, weighted for the current strategy frequencies. Formally, the expected new population size for strategy *j* is given by:

3 $$\begin{aligned} N_j^{t+1}= N_j^t + \left( N_j^t\cdot \overline{ P^t_j}  - N^t_j\cdot \overline{P_t}\right) \delta \end{aligned}$$

where $\overline{P^t_j}$ is the average payoff of strategy *j*, while $\overline{P_t}$ is the average payoff in the whole population and $\delta$ is the speed of evolution.^4^ We constrain the finite replicator dynamics process such that all strategies are represented by an integer number of agents in the population and no $N_j^{t+1}<0$. Note that the strategies of agents evolve and not the agents are replaced. This means that agents accumulate editorial and public reputation throughout the simulation.

### Reputation-based selection and journal impact factor

In order to keep the model simple and to concentrate on some key mechanisms, other details are fixed to some natural values and some possible elements can be activated upon choice, which we list below.

*Limit to the number of publications* The number of publications is limited to a fixed proportion $\epsilon < 1$ of submissions. If the referee process produces too many accepted papers given the current editorial policy of the editor, then all accepted contributions are ranked according to editorial reputation $REP_i^E$ of the authors and only the first $\epsilon N$ papers are published. In scientific practice, journal rejection rates vary largely between journals and disciplines. Rejection rates might be lower in the physical sciences and larger in the social sciences and humanities (Zuckerman and Merton 1971; Hargens 1988). In most reported simulations, we opted for a more conservative value of $\epsilon =0.3$ that is closer to the latter. We varied the publication rate from zero to hundred percent in additional simulation runs (reported in the “Results” section, Figs. 4, 5, 6).

*Journal impact factor* In recognition of the fact that publishing in a reputed journal produces a payoff that depends also on the quality of past published papers, agents who publish a paper may receive an increase to their payoff equal to:

4 $$\begin{aligned} JIF_t=\kappa \cdot \frac{\sum _{\tau =1}^t \sum _{i=1}^N GP_{i}^{\tau }}{\sum _{\tau =1}^t \sum _{i=1}^N GP_{i}^{\tau } + \sum _{\tau =1}^t \sum _{i=1}^N BP_{i}^{\tau }} \end{aligned}$$

At each given time step *t*, the public good benefit of journal impact factor (JIF) is given to all authors who get their papers published, irrespective of quality. That is, a public good bonus is added to the payoff of each agent publishing a paper. The size of the public good is proportional to the performance of the journal in terms of the proportion of high-quality papers published up to that point in time. The higher the proportion of good papers, the higher the JIF is. Note that the introduction of JIF increases payoffs for publications produced at high effort, but increases free rider rewards for those who are able to publish low-quality work to the same extent. We assume that the journal impact factor is a public good for those who published with a linear production function, where the increment $\kappa \ge 1$ describes the public value of a single scientific contribution. The lack of a journal impact factor and a linear public good with $\kappa \le 1$ describes a situation that is worse than the Prisoner’s Dilemma: even full cooperation does not compensate for the entailed costs. Considering the journal impact factor and $\kappa >1$, translates the game into a true linear Public Good Game, which is still extremely difficult to solve. In the illustrative runs, we fix $\kappa =2$, but we show in the robustness checks reported in Fig. 15 in the “Appendix” that our results do not change as long as JIF actually is a true public good multiplier ($\kappa >1$).

*Desk-rejections and speeding up publication* With increased time-pressure and burden of reviewers, it is common practice that not all submissions are sent out for review. Some submissions are desk-rejected and others have an easy, speedy route for publication. Editorial decisions behind this behaviour are very much based on the reputation of authors. We implement desk-rejection and desk-acceptance as an additive feature compared to the baseline model.

Desk-rejection and acceptance introduces a bias in favour of individuals with higher reputation and in damage of individuals with lower editorial reputation compared to the baseline model. Essentially, we introduce the possibility for the editor to desk-reject or accept submissions proportional to the relative editorial reputation of the author, regardless of recommendations by referees. Please note that as producing low or high-quality reviews is also part of editorial reputations, in this way, reviewers receive some compensation for their low or high reviewing efforts. The rule added to editorial decisions is as follows:

- Compute the minimal editorial reputation $min(REP_i^E)$, the maximal editorial reputation $max(REP_i^E)$ and the median editorial reputation $median(REP_i^E)$ of agents.
- If $REP_i^E<median(REP_i^E)$, then with probability
  5 $$\begin{aligned} P_{dr}=\frac{REP_i^E - min(REP_i^E)}{median(REP_i^E)-min(REP_i^E)} \end{aligned}$$
  the paper is sent out for review, and with probability $1- P_{dr}$, it is desk-rejected.
- If $REP_i^E>median(REP_i^E)$, then with probability:
  6 $$\begin{aligned} P_{da}=\frac{REP_i^E - median(REP_i^E)}{max(REP_i^E)-median(REP_i^E)} \end{aligned}$$
  the paper is desk accepted, and with probability $1- P_{da}$, it is sent out for review.

## Results

### Baseline results

**Fig. 1:**
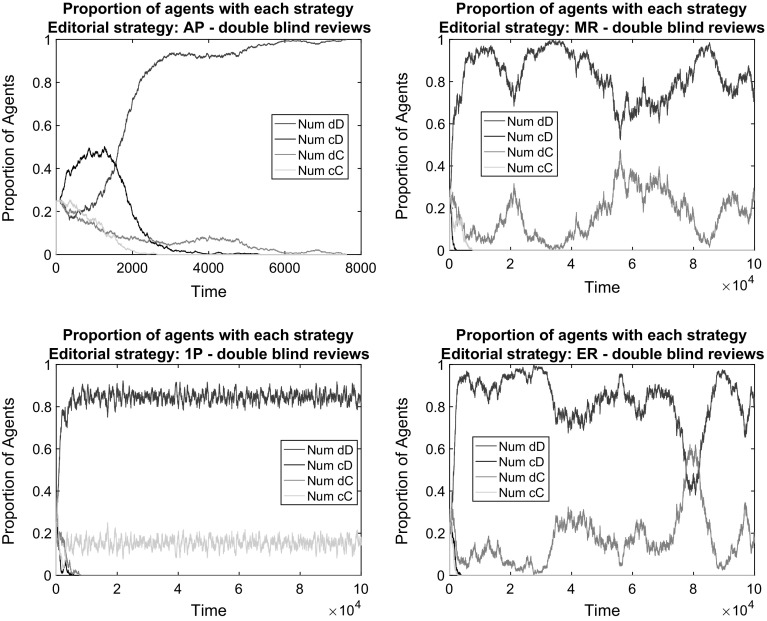
The failure of scientific cooperation in the baseline model with double blind reviews (2 random referees) under different editorial policies. Respectively: AP (*top left*), MR (*top right*), 1P (*bottom left*), ER (*bottom right*). *Notes:* All illustrative simulations concern the baseline model: the population is composed of 1200 individuals; initially divided among the four possible bundled strategies; simulations run till convergence or until $t_{max}=100{,}000$; papers are assigned in random order to randomly chosen referees; each referee can review up to 4 papers and a maximum of 30% of the papers is publishable by space limitations

**Fig. 2:**
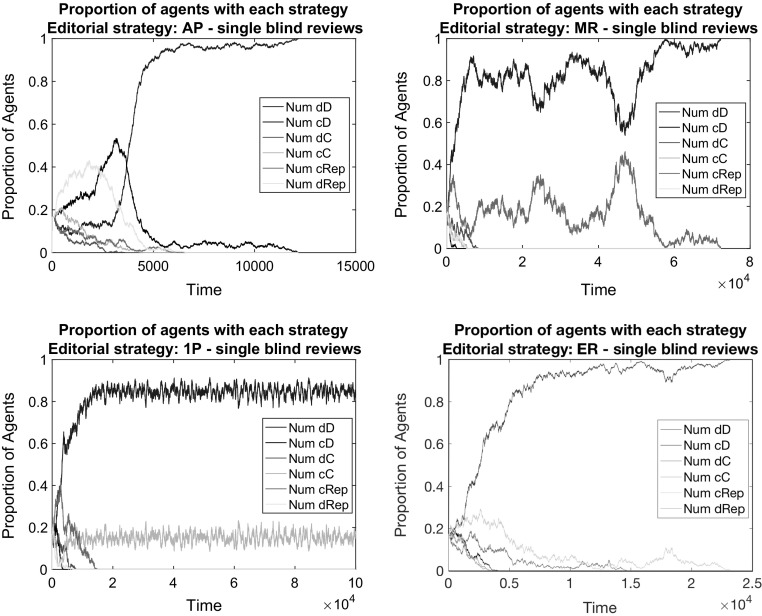
The failure of scientific cooperation in the baseline model with single blind reviews (2 random referees) under different editorial policies. Respectively: AP (*top left*), MR (*top right*), 1P (*bottom left*), ER (*bottom right*). *Notes:* All illustrative simulations concern the baseline model: the population is composed of 1200 individuals initially divided among the six possible bundled strategies; simulations run till convergence or until $t_{max}=100{,}000$; papers are assigned in random order to randomly chosen referees; each referee can review up to 4 papers and a maximum of 30% of the papers is publishable by space limitations

We first demonstrate that in the baseline model in which scientists face costs for producing high-quality manuscripts and costs for producing high-quality reviews, there is almost no chance of any cooperation. This is not surprising, because low effort in writing papers as well as low effort in writing reviews is the dominant strategy in the baseline game. Figures 1 and 2 report that this is the case both for double blind and for single blind peer review systems considering a neutral editorial policy (AP).^5^ In all cases, the strategy implying the production of low quality papers and reviews (dD) overtakes the entire population. To examine the failure of the scientific peer review process more closely, consider that high-quality review does not return any benefits, therefore every reviewer is better off by choosing D. In a population with only dD and cD strategies, dD yields higher average payoffs since, if people review randomly, there is a 50 % chance of getting a low-quality paper published, which is exactly the same for high-quality submissions. As cD strategies do not benefit anything from peer review and they entail higher costs for the author; they die out. Without any feedback loop that would help to ensure the production of scientific quality, science ends up as an empty exercise.

**Fig. 3:**
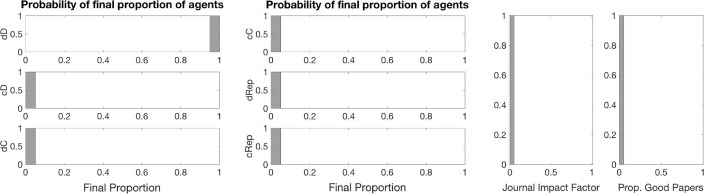
Baseline distribution of each strategy type (*left and central panels*) and journal statistics (*right*) at the end of simulation. *Notes:* Results are distributions from 100 iterations with editorial strategy AP. For all simulations, we considered single blind review; papers assignment in random order to two random referees; a population of 1200 individuals initially divided among the six possible bundled strategies; each referee can review up to 4 papers; and a maximum of 30% of the papers is publishable by space limitations. Simulations run till convergence or until $t_{max}=100{,}000$

*Baseline with reputation-weighted consideration of reviews* Figures 1 and 2 contain also cases in which the editorial policy takes into account the editorial reputations of reviewers and attach higher weights to the opinion of higher reputed referees. These are editorial policies MR and ER. The strategy dD still gains overwhelming dominance under these policies. This is because such policies do not ensure the occurrence of correlated equilibria: the larger weight to opinions does not mean at all that these opinions would favor high-quality contributions more than others. Low effort in reviewing is a dominant strategy for every author. The opinion of those who started with cooperation receive higher weights, but they still underscore defectors with regard to payoffs. The initial population that is equally divided among different types of strategies goes through a quick and progressive elimination of cooperative strategies. In this process, having a relatively bad reputation does not matter for payoffs as the chances of publishing a paper become equivalent for good and bad papers over time.

*Baseline with the entry of reputational concerns* Somewhat surprisingly, the editorial policy that ensures a low, but stable level of cooperation is 1P. This is an editorial policy, which accepts all papers, if there is at least one positive recommendation from the reviewers. The surprising result originates in the lack of cooperation of reviewers. When reviewer recommendations are random, it should not matter from the quality point of view, how many reviewers have suggested the given manuscript for publication. In case of two reviewers and editorial policy AP, both reviewers need to recommend publication, which will happen in approximately 25% of the cases. This is within the limits for publication ($\epsilon =0.3$), hence the editor does not need to rely on individual reputations.

As there is a constraint on how many papers can be published, in case of random reviews, the friendly 1P editorial policy allows—in principle—publication of approximately 75 % of the submissions. In case of a surplus, the editor ranks the papers based on the editorial reputation of authors. Hence, a direct feedback on reputation exists, which is sufficient to guarantee in some simulation runs a stochastic mixed strategy equilibrium with the survival of cooperation.

### Reputational concerns and increased publication rate

**Fig. 4:**
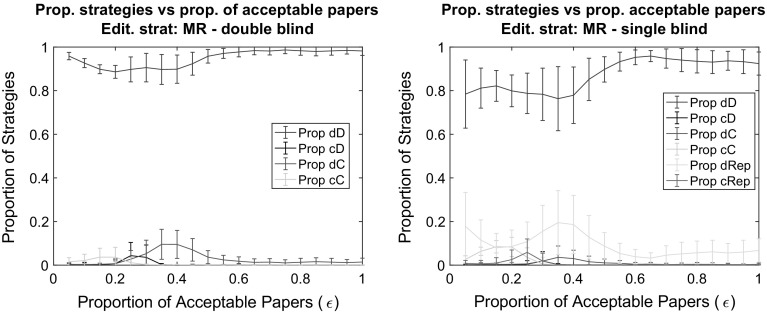
The impact of the proportion of acceptable papers ($\epsilon$) in a double blind (*left*) and single blind (*right*) peer review system on the proportion of strategies at the end of the simulation. *Notes:* Editorial strategy is MR. Other parameters are as in the baseline (see Fig. 3)

We varied the publication rate from zero to hundred percent in additional simulation runs for editorial policies MR and 1P, using both single and double blind reviews. For the MR case, we did not find any significant differences compared to the baseline cases (Fig. 4) in line with what discussed in the previous section. Irrespectively of the publication rate, the dD strategy has overwhelming success: scientists place low effort in their reviews as well as in their work.

**Fig. 5:**
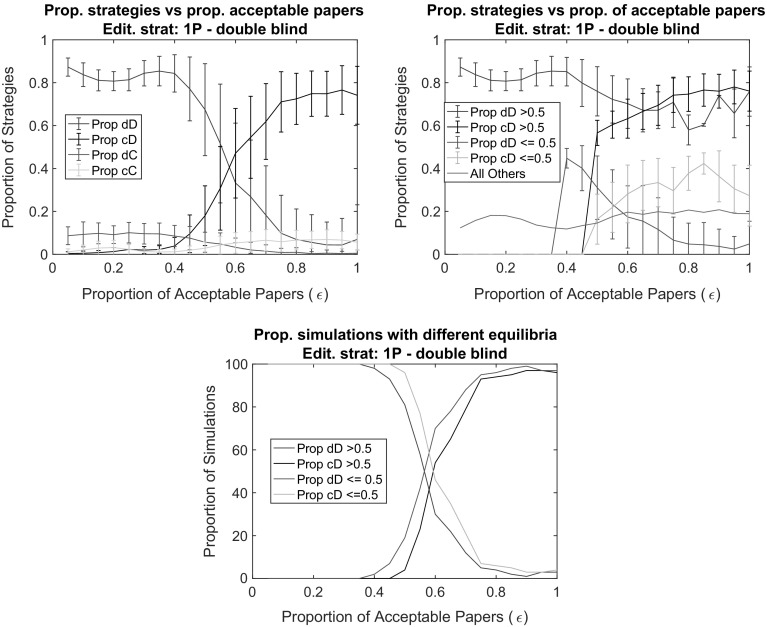
The impact of the proportion of acceptable papers ($\epsilon$) in a double blind peer review system on the proportion of strategies at the end of the simulation (*top left*). To provide more detail, the *top right panel* separates simulations in which the dD strategy has gained majority from those in which the cD strategy gained majority. The *bottom panel* reports results for the proportion of simulations runs in which the majority strategy has values above and below 0.5. *Notes:* Editorial strategy is 1P. Other parameters are as in the baseline (see Fig. 3)

**Fig. 6:**
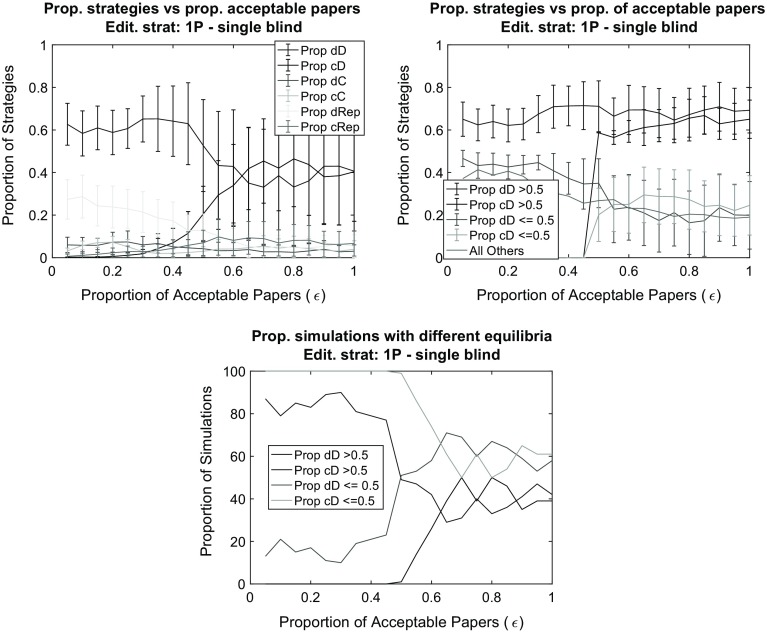
The impact of the proportion of acceptable papers ($\epsilon$) in a single blind peer review system on the proportion of strategies at the end of the simulation (*top left*). To provide more detail, the *top right panel* separates simulations in which the dD strategy has gained majority from those in which the cD strategy gained majority. The *bottom panel* reports results for the proportion of simulations runs in which the majority strategy has values above and below 0.5. *Notes:* Editorial strategy is 1P. Other parameters are as in the baseline (see Fig. 3)

Again, the mild editorial policy 1P provides the better avenue for high efforts in writing papers. For the editorial policy 1P, we found a higher level of cooperation by increasing acceptance rate (Figs. 5, 6). That is, with the increase of journal space, simulations were more likely to end with a success of the cD strategy. Scientists place high effort in their work under conditions when it was easier to get it published. This is a very important result: cooperation is established with a “mild” editorial policy and in a “mild” environment more likely than in the case of strict editorial policy and a competitive environment.

The surprising result originates from the role of rejections in the editorial reputation calculation. When nearly all papers are published, the true qualities of nearly all papers are revealed. Bad papers written with low effort result in a loss of editorial reputation, which weighs largely for scientists’ publication chances. The power of publicity is not there in a competitive environment where $\epsilon$ is lower. Given the random reviewer recommendations, good papers are rejected just as likely as bad papers and therefore imply costly rejections for high effort authors. Hence, under such circumstances, low effort in writing papers is the ultimate beneficial strategy.

### Reputation bias in editorial policy

As we demonstrated, accounting on reputation of reviewers for making judgment on manuscripts is insufficient to trigger the production of high-quality reviews and high-quality papers. We shall see if a more direct consideration of editorial reputation leads to higher efforts and as a result to better science. Note that the model extension in this direction is related intentionally to popular discussions whether editors should be unbiased or they could rely on reputation signals of authors from the past. Should they catalyze the Matthew effect in science, in which the successful get even more success (Merton et al. 1968; Squazzoni and Gandelli 2012)? Should they contribute to the maintenance of the old-boyism bias? If they do, does it hurt or help scientific development?

Let us first introduce an editorial bias in favour of authors with high $REP_i^E$ and against authors with low one.^6^ We assume that authors with an editorial reputation lower than the median has a chance of desk rejection that is in negative linear association with their reputation (Eq. 5). Similarly, we assume that authors with an editorial reputation higher than the median has a chance of desk acceptance that increases linearly with their reputation (Eq. 6). This modification does not rule out peer review, but concentrates its decisive character to the middle range, where no clear reputational judgment can be expected from the editor.

**Fig. 7:**
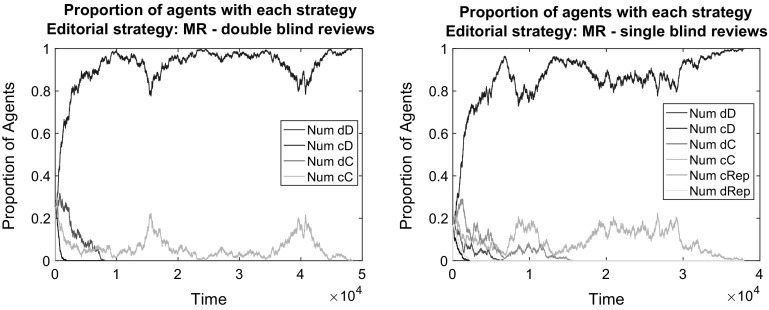
The impact of reputation bias in editorial policy in desk acceptance and rejection of papers: double blind review (*left*) and single blind review (*right*). *Notes:* Papers are assigned in random order to two random referees. The editorial strategy is MR. Other parameters are as in the baseline

**Fig. 8:**
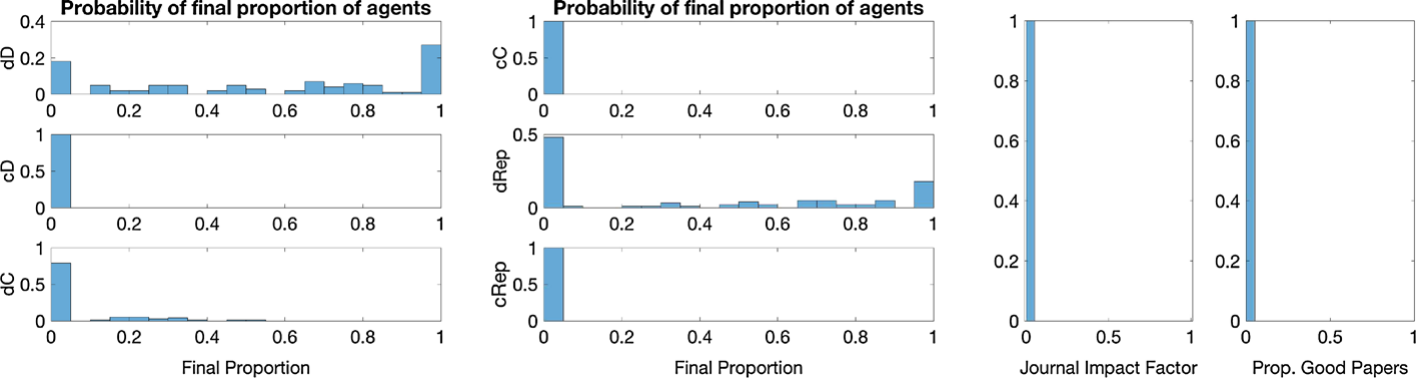
The effect of using reputation in editorial policy. Final distribution of each strategy type (*left and central panels*) and journal statistics (*right*) at the end of simulation. *Notes:* Results are distributions from 100 iterations with editorial strategy AP and a single blind review system. Other parameters are as in the baseline (see Fig. 3)

Results with this extension show no major breakthrough for cooperation: dD dominates the outcomes (Fig. 7). Either with double blind or single blind peer review, all agents become of dD types. This indicates that a direct editorial bias in desk acceptance and rejection in itself is insufficient to trigger a large extent of cooperation. This kind of editorial bias, however, is able to support the survival of conditional cooperation of reviewers (Fig. 8). When a strict editorial acceptance policy is applied (AP), the lack of publishable material leads to the need of selecting submissions based on reputation. The high effort in producing scientific material, however, does not pay off because of the difficulties of acceptance. Scientists therefore follow the easier path of gaining higher reputation and might place high effort in reviewing others. Reviewing efforts are profitable when they are most likely to provide reputation benefits. The public reputation helps the referees to get the best out of their reputation-based conditional strategy: when the public reputation of an author $REP^P_i$ is high, then it is more likely that his paper gets published, and therefore it is more likely that a review of high quality will ensure positive returns in terms of editorial reputation. As a result, the cooperative reviewer strategy that is conditional on the reputation of the author might survive in case of single blind peer review. This happens because the editor might provide a differential treatment for individuals with higher reputations earned strictly by high-quality reviews. At the opposite, when the author’s reputation is low, then reviewers with a strategy conditional on author reputation do not bother and follow the cheap strategy of providing random advice. In this case, their payoff is not different from agents who never put high effort in reviewing.

### Introducing the public good of journal impact factor (JIF)

**Fig. 9:**
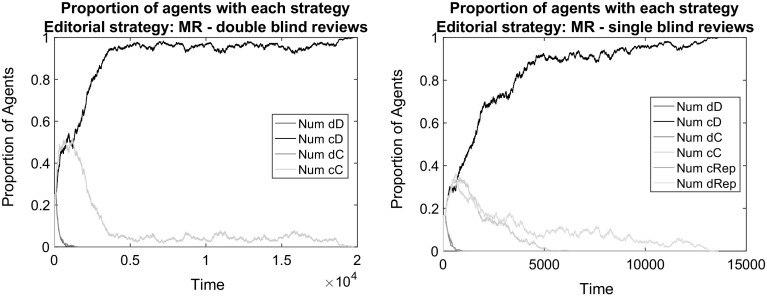
The effect of introducing a journal impact factor into the payoffs: in a double blind (*left*) and single blind (*right*) peer review system. *Notes:* Papers are assigned in random order to two random referees. Editorial strategy is MR. Other parameters are as in the baseline

Large public good benefits in the presence of some reputational motives allow for strategies producing high quality papers to survive and disseminate (Fig. 9). Furthermore, the analysis of the population evolution shows that when JIF is active, most strategies producing low-quality papers disappear from the population. This means that if a journal publishes high-quality papers, it ensures that future submissions will also be of high quality.

**Fig. 10:**
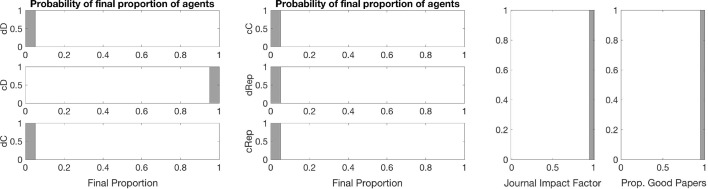
The effect of accounting journal impact factor into the payoffs. Distribution of each strategy type (*left and central panels*) and journal statistics (*right*) at the end of simulation. *Notes:* Results are distributions from 100 iterations with editorial strategy AP and a single blind review system. Other parameters are as in the baseline (see Fig. 3)

This is good news given the fact that the public good reward of JIF as a payoff supplement does not erase the social dilemma structure of the game. Defection is still the best reply strategy both for authors and for reviewers. Still, cooperation evolves; thanks to the editorial account of author reputations and to the large initial share of cooperative strategies that survive the early phase of the simulation. Full cooperation is among those who disappear relatively late (Fig. 9), which assists the dominance of the high-effort-in-writing and low-effort-in-reviewing strategy. As a consequence, the rise of good papers at the end is not accompanied by good reviews (see Fig. 10 for a statistical assessment). Still, the scientific development is maintained and results in the highest possible JIF. This means that only high quality papers are published. Peer review just adds a random noise for the publication process and it is meaningless anyway because everyone contributes with high-quality submissions.

*Journal impact factor together with strong reputation concerns* Public good rewards that supplement the original payoff structure largely improve the opportunities for scientific development and lead to the overall success of the cD strategy. We have also seen that under certain editorial policies that take account of reputation, a low level of full cooperation (cC) can be sustained also without the public good reward. When we introduced desk rejections and acceptance based on reputation to the baseline, then some agents gained reputation successfully with a conditional reviewing strategy dRep. It is therefore interesting to observe which strategies are successful if both JIF and strong reputation concerns are accounted for.

**Fig. 11:**
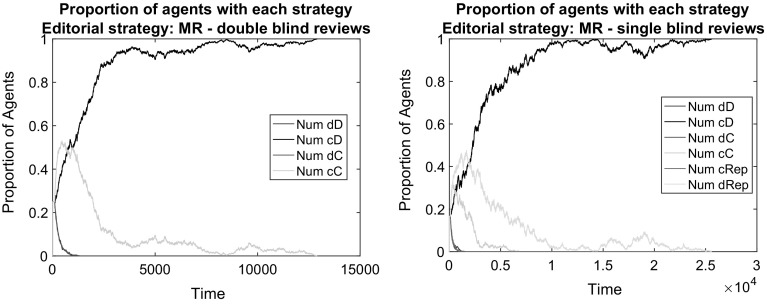
The effect of accounting journal impact factor into the payoffs and using reputation in editorial policy: in a double blind (*left*) and in a single blind (*right*) peer review system. *Notes:* Papers are assigned in random order to two random referees. Editorial strategy is MR. Other parameters are as in the baseline

**Fig. 12:**
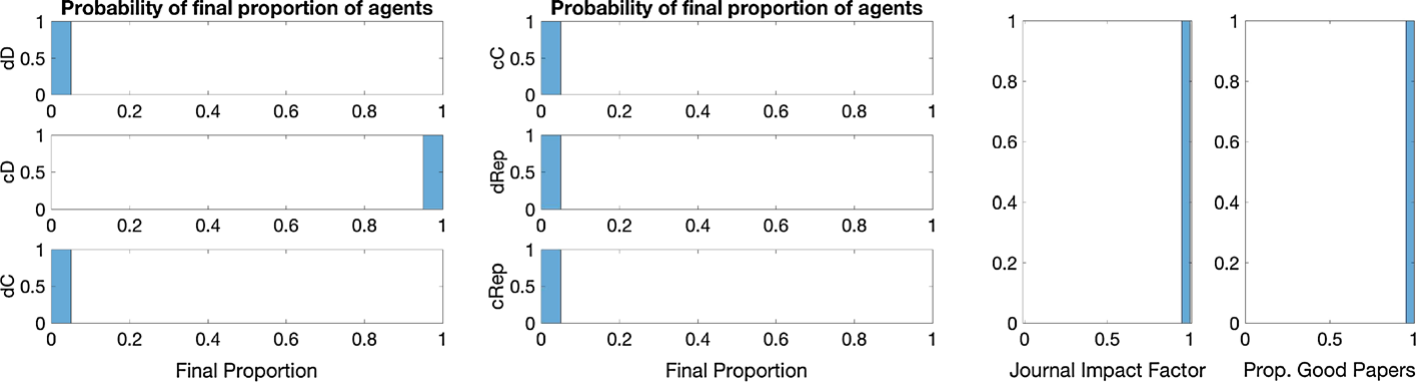
The effect of accounting journal impact factor into the payoffs and using reputation in editorial policy. Distribution of each strategy type (*left and central panel*) and journal statistics (*right*) at the end of simulation. *Notes:* Results are distribution from 100 iterations with editorial strategy AP and single blind review system. Other parameters are as in the baseline (see Fig. 3)

**Fig. 14:**
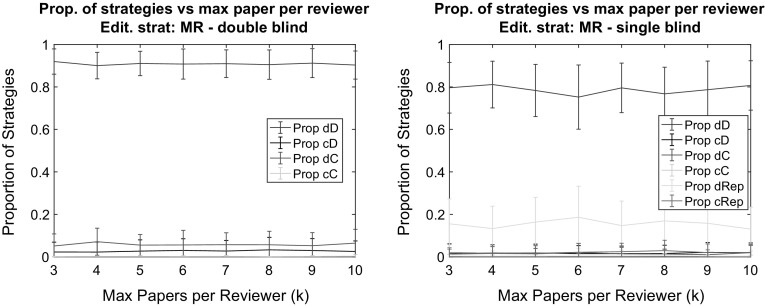
The impact of the journal impact factor multiplier in the payoffs ($\kappa$) in a double blind (*left*) and single blind (*right*) peer review system on the proportion of strategies at the end of the simulation. JIF multiplier values of $\kappa$ are explored from 0.8 to 4 in steps of 0.4. *Notes:* Papers are assigned in random order to two random referees. Editorial strategy is MR. Other parameters are as in the baseline (see Fig. 2)

**Fig. 15:**
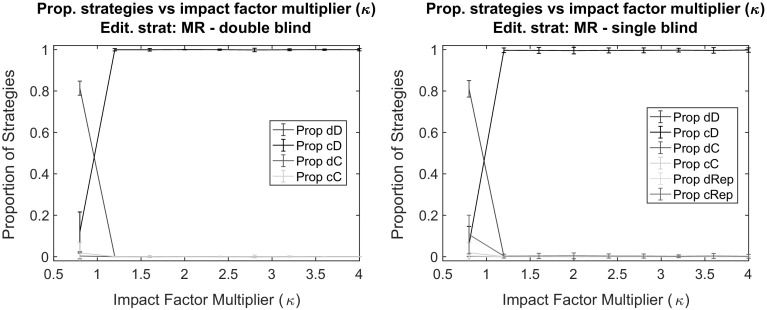
The effect of using **public** reputation in editorial policy in a double blind (*left*) and single blind (*right*) peer review system. *Notes:* Papers are assigned in random order to two random referees. Editorial strategy is MR. Other parameters are as in the baseline (see Fig. 2). Results are in *line* with those of Fig. 7

The results show that the strongest determinant of the evolution is the journal impact factor (Figs. 11, 12). When it matters, even under strong reputation concerns, high-effort publication strategies gain dominance with low-effort reviews. This is very much meaningful once there is a reward for reputation and the most reputational gains can be obtained by high-quality publications.

*Tacit agreements* To complete the story, we still miss a mechanism that makes reviewing as well as writing papers plausible and sustainable without radically altering the payoff structure of the game. A realistic possibility is to consider “tacit agreements” that are based on direct reciprocity and might work better in case of single blind reviews, in which at least one side of the informational asymmetry is relaxed. “Tacit agreements” work against the impartiality of peer review in practice. A “nice” tacit agreement strategy could start with high effort in the first *n* rounds, then recognize previous reviewers of own papers with *p* probability and retaliate them not with high effort/low effort, but with acceptance/rejection recommendation. Once there is a coordination device that brings high quality submissions in the hand of highly reputed reviewers, then acceptance recommendations would match true quality. In this case, the editorial selection policy will matter, because this would create the possibility of low cost reciprocation for high performing scientists. This is in line with the conclusion of similar empirical and simulation work (such as Bianchi et al. 2017). Some scientists believe that top researchers in top journals are involved in such reciprocity. It is important to note the possible drawbacks of such self-emerged network-based practices, including old-boyism, partiality, and the conservative bias (Sarigöl et al. 2014; Sobkowicz 2015; Soós et al. 2015).

## Conclusion

It is puzzling why scientists devote considerable time and effort for writing reviews that decreases their time spent on their own research. Once everyone acts according to self-interest, reviews are all of low effort and they cannot be adequately used to judge scientific quality. As a consequence, scientists submit low quality work in the hope of passing through randomly judging reviewers. Still, in practice, reviewers as well as authors invest high effort in reviews and submissions. We have labeled this puzzle as the miracle of peer review and scientific development. We investigated some potential mechanisms that might resolve the puzzle in an agent based model.

We have modeled scientific production accordingly: with an incentive structure in which low effort in writing papers as well as in writing reviews is the dominant strategy of agents. We applied a replicator dynamics rule to the population of scientists, allowing for the reproduction of strategies that result in higher payoffs. Not surprisingly, in our baseline model, low effort in writing papers as well as in writing reviews have spread in the population and scientific practice has become an empty exercise.

Next, we assumed that editors might rely on the reputations of authors in their choices. In our model with a single journal, the editor took perfect account of high- and low-quality publications of authors, the number of their rejected papers, and if their reviewer recommendations were in line with the true quality of the paper or not. We examined different editorial policies that took account of the reputations of scientists. We showed that if reputations are used in editorial assessment of reviewers’ suggestions, then it does not save science from low quality submissions and low quality reviews. A bit more surprisingly, easing the route for publication by desk acceptance for highly reputed authors alone has not changed anything either. All this indicated that the emergence of cooperation in the form of high efforts is a difficult motivational puzzle.

Some cooperation has resulted from a friendly editorial policy that categorized submissions as publishable if at least one reviewer recommended publication. This policy led to an oversupply of publishable material, which called for the ranking of submissions based on author reputations. This direct feedback has made the investment in reputations profitable. Consequently, high-effort author strategies survived in a mixed equilibrium together with low-effort author strategies, but nobody invested effort in reviewing.

Some cooperation has emerged also when a strict editorial acceptance policy was applied, in which only papers with unanimous reviewer support are published. In this case, however, the lack of publishable material was responsible for the worth of reputation. As the investment in reputation via writing papers was risky due to the difficulties of publishing, scientists profited more from the investment in reputation via reviews. A strategy that conditioned high reviewing efforts on the author’s reputation was able to gain a notable share in the population.

Reputations worked to some extent, but public good benefits were clearly better catalysts of scientific development. Once we introduced the journal impact factor as a public good benefit, which meant the distribution of an additional payoff for all authors who published in the journal (either a good or a bad paper), cooperation has become the most successful strategy of authors. In this case, editorial reputations became correlated with actual contributions to the provision of the pubic good. But as the production of high quality papers was still much more important for reputations than high quality reviews, the cooperative strategy that emerged as successful was investing high effort only in manuscript writing and not in reviewing. As a consequence, cooperation has been observed in scientific production, but peer review has just added random noise to this development, which raises doubts of its use (in line with Neff and Olden 2006) and concerns about the use of public money for this activity.

At the end, we were successful in demonstrating in a simple model the puzzling motivational problem of peer review. We highlighted that it is not easy to find the way out of this puzzle. We showed that a high value of the public good of science maintains scientific development. Reputational systems that are heavily building on author contributions might be partially sucessful, especially if the reputational hierarchy is directly used for selecting between similarly rated submissions. These mechanisms, however, will not help to sustain the efficiency of peer review. Paradoxically, mechanisms that are able to induce some level of high-quality reviews are building on reciprocity. In practice, they are often associated with partiality, old-boyism, the emergence of invisible colleges, the Matthew effect, the conservative bias, and the stratification of science.

Future work should extend our simple model towards studying multiple journals that compete for success with each other. This extension would allow for the evolution of editorial strategies in a straightforward way and in parallel to theoretical studies that highlight how group selection can ensure higher cooperation (Traulsen and Nowak 2006; Nowak 2006), is expected to lead to better reviewer performance. Our current study and subsequent research on the social dilemma of peer review might help us to understand how the evaluation system of scientific work can be sustained. Once the fundamental mechanisms are studied rigorously, they can also lead to policy recommendations on improvements of the current system and the design of new solutions (cf. Paolucci and Grimaldo 2014).

## Figures and Tables

**Fig. 13:**
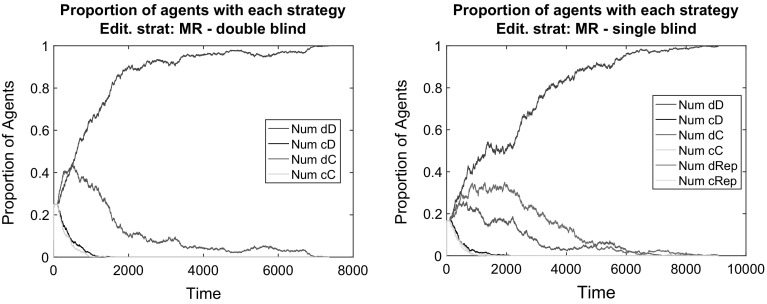
The impact of the maximum number of papers that a reviewer is willing to accept per period (*k*) in a double blind (*left*) and single blind (*right*) peer review system on the proportion of strategies at the end of the simulation. Values of $\kappa$ are studied from 3 to 10. *Notes:* Papers are assigned in random order to two random referees. Editorial strategy is MR. Other parameters are as in the baseline (see Fig. 2)

## Appendix: additional simulations and robustness checks

See Figs. 13, 14 and 15.

## Footnotes

1 Alternative specifications, where editorial decision concerning the referees to assign to a paper are proportional to the reputation of scientists and/or where the editor assigns reviewers first to the papers of most reputed individuals have been studied. In all cases, results qualitatively are in line to those reported in the “Results” section, thus results are not reported for space issue.

2
$E=0.1$ and $e=1$ in the simulations reported.

3 This option is only available for the single blind case.

4 For the sake of our simulations, the parameter $\delta$ is set to 0.0004, this very conservative value implies a slow evolution of our model.

5 Figure 3 shows that the results is confirmed for editorial policy AP when 100 iterations of the setup are run. For other editorial policies, statistical results are in line with those of the representative simulation reported.

6 As shown in Fig. 13, the same results are obtained assuming that the editor adopts the public reputation $REP_i^P$ as meter for bias in editorial behaviour.
